# Facilitating knowledge transfer to policy makers and front‐line workers during a pandemic: Implementation, impact and lessons learned

**DOI:** 10.1111/hir.12523

**Published:** 2024-03-11

**Authors:** Nicola Pearce‐Smith, Emma Farrow, James Robinson, Blathnaid Mahon, Cat McGillycuddy, Kester Savage

**Affiliations:** ^1^ UK Health Security Agency (UKHSA) London UK

**Keywords:** collaboration, critical incident, current awareness services, human‐computer interaction, impact, public health, selective dissemination of information (SDI), surveys

## Abstract

**Background:**

Stakeholders working on the COVID‐19 pandemic response needed access to evidence, requiring a systematic approach to identify and disseminate relevant research.

**Objectives:**

Outline the stages of development of a COVID‐19 Literature Digest; demonstrate the impact the Digest had on decision‐making and knowledge gain; identify the lessons learned.

**Methods:**

A standardised process was developed to identify and select papers. The main sources for content were PubMed, bioRxiv and medRxiv. A shared EndNote library was used to deduplicate and organise papers. Three user surveys obtained feedback from subscribers to determine if the Digest remained valuable, and explore the benefits to individuals.

**Results:**

40–60 papers were summarised each week. 211 Digests were produced from March 2020 to March 2022, with around 10,000 papers included altogether. Survey results suggest benefits of the Digest were gaining new knowledge, saving time and contributing to evidence‐based decision making.

**Discussion:**

Digest procedures constantly evolved and were adapted in response to survey feedback. Lessons identified: learn from failure, communication is key, measure your impact, work collaboratively, reflect and be flexible.

**Conclusion:**

The Digest was successfully produced within the limits of available resource. The learning from this Digest will inform evidence monitoring, selection and dissemination for future health crises.

**Key Messages**

- Rapid creation and dissemination of an evidence digest is a valuable service during a pandemic as it provides stakeholders with essential knowledge and saves them time.
- These instructions for rapidly and sustainably producing an evidence digest should facilitate librarians to develop future timely good quality products.
- Before producing a regular evidence digest, librarians should create a living Standard Operating Procedure to enable standards and quality of the product to be maintained.
- The survey findings suggest that the public health workforce used the digest to reduce information overload and appreciated being reliably informed.

## BACKGROUND

On 31 December 2019, a cluster of cases of pneumonia was reported in China; the World Health Organization declared the coronavirus disease 2019 (COVID‐19) outbreak a global pandemic on 11 March 2020. In times of emergency response, front line workers and policy makers need access to evidence as it emerges to inform response and mitigation activities, and this required a systematic approach to identify emerging evidence by our public health agency. The COVID‐19 Literature Digest (herein known as the ‘Digest’) was established in February 2020 to disseminate timely, relevant evidence to stakeholders working on the Public Health England (PHE)—later, UK Health Security Agency (UKHSA) (UK Health Security Agency, 2021)—COVID‐19 pandemic response.

There was an overwhelming availability of new research evidence from a myriad of sources (Teixeira da Silva et al., 2021), in peer‐reviewed journals, organisational websites and preprint servers (posted without peer review). Early studies often lacked control groups or enrolled too few people (Pearson, 2021), which also lead to generation of poor‐quality systematic reviews. The quality of this evidence was very variable (Glasziou et al., 2020; Jung et al., 2021) and required critical appraisal/review by technical experts before being incorporated into evidence informed clinical or public health guidance and policy.

COVID‐19 research was not always published in mainstream journals or indexed in medical databases such as Medline, so standard database searching was not sufficient. New COVID‐19 websites and resources emerged, such as COVID‐19 L·OVE (Epistemonikos, 2020) and LitCovid (National Library of Medicine, 2020).

Many research papers were posted on preprint servers as authors looked for a way of rapidly disseminating their work. Preprints: ‘preliminary reports of work that have not been certified by peer review’ (Cold Spring Harbor Laboratory et al., 2020b) were shared before the COVID‐19 pandemic, but predominantly by physicists and mathematicians (e.g., on ArXiv). The rise in the number of COVID‐19 preprints has been substantial (Callaway, 2020), and has seen servers such as medRxiv and bioRxiv create specific collections of COVID‐19 preprints (Cold Spring Harbor Laboratory et al., 2020a). The first COVID‐19 preprint was posted on bioRxiv only 22 days after cases were discovered in China (Brierley, 2021).

Findings from poor quality COVID‐19 preprint studies were often reported in the media as confirmed fact, especially studies reporting novel results (Glasziou et al., 2020). For example, a paper uploaded to a preprint server on 1 July 2020, which claimed to find an association between COVID‐19 mortality and low levels of Vitamin D was downloaded more than 17,000 times and mentioned in social media over 8000 times, but the existence of the Indonesian authors could not be confirmed and the preprint was later removed—by then, misinformation had been spread via the media and cited by several publications (Henrina et al., 2021).

It was necessary to develop a sustainable process to find, assess, select and categorise relevant COVID‐19 research from these sources into a report to provide stakeholders with a selection of timely, summarised papers which were relevant to UK settings, and contained new findings, insights or emerging trends. Decision makers needed to focus on the content and not the searching process; and the team providing the Digest needed to manage the quantity of literature. No existing open source compilation of information was available in February 2020, which necessitated the development of the Digest. The Digest was produced by individuals with either library and information skills or those with public health experience.

## OBJECTIVES

The main objective of this article is to outline the stages of development and production of the Digest, including the changes and improvements made to the process during the last two years, and to demonstrate the impact of the Digest on decision‐making and knowledge gain. The article aims to provide both a detailed instruction manual and a lessons learned document, in order to inform those who may be required to rapidly disseminate evidence in the future.

## METHODS

### Identifying and selecting evidence for the Digest

In order to highlight a selection of COVID‐19 research, a process was required to identify, assess and select papers. The main sources for content were PubMed and the preprint platforms bioRxiv and medRxiv. PubMed was selected as it is the most up‐to‐date way of accessing Medline. medRxiv and bioRxiv were identified as preprint sources most likely to contain relevant papers; other sources containing preprints such as aRxiv were rejected as they seldom contained relevant or unique papers. Some journals were searched directly from the publisher website in order to capture new papers that were not yet indexed on PubMed. Later, we were able to select only the key journals (identified by calculating the number of articles from each journal included in our Digest between January 2021 and August 2021) for this process.

Grey literature sources were also searched—for example, the GOV.UK daily email updates for government reports and non‐peer reviewed papers presented to Scientific Advisory Group for Emergencies (SAGE) committee (Sage (Scientific Advisory Group for Emergencies) Committee, 2022). Other current awareness products were also scanned e.g. email alerts from Johns Hopkins University and the Canadian Public Health Agency; and media sources like the BBC (BBC, 2022) for press releases about new research.

Citations obtained from the searches were screened using a standard operating procedure (SOP), with inclusion/exclusion criteria covering relevance, quality, type of research and novelty. The SOP contained instructions for all production stages of the Digest, and continued to develop as methods improved, inclusion criteria became more focused and processes were streamlined. This living SOP document was essential to maintain standards within the Digest, especially when several staff were involved in the production. Selected papers were categorised by theme with a short bullet point summary added. Themes were tailored in response to current topics appearing in the literature, the needs of UKHSA COVID‐19 response staff, and from suggestions obtained in user surveys.

Initially, the Digest references were managed using a Microsoft Excel spreadsheet, but with the increasing number of research papers available, and the necessity to group, search, deduplicate and share results, a new solution was needed. EndNote reference management software (Clarivate, 2022) became the platform used to sort, deduplicate and organise papers. The ability to create a Shared Endnote library was crucial when up to four members of the Digest team were working simultaneously. The shared Endnote library required staff to synchronise (Sync) any local changes in order to update the library held on a web‐based Cloud.

PubMed and the preprint platforms were searched daily using predetermined search strategies, and citations imported into the shared Endnote library, which was set to automatically deduplicate on import. Searches in PubMed were run at least twice a day due to the volume of literature available, the frequency that PubMed was updated and the time‐difference between United Kingdom and United States.

### Streamlining of screening process

The Smart Group library as a new first step for screening PubMed papers was introduced in early 2021. Groups are defined by Clarivate as ‘a subset of references that already exist in the library’. Smart Groups: ‘are built with search strategies. Smart Groups are dynamically updated as you add references to and edit references in the library’. When citations are imported or copied into a Library containing Smart Groups, those matching the Smart Group terms are automatically moved into the relevant folder(s).

We created search strategies for 12 Smart Groups for each of our major COVID‐19 themes, with some additional Smart Groups using the Author Address field to capture papers containing UK or United Kingdom, and one containing Public Health England (and later UK Health Security Agency and UKHSA). Over time, extra Smart Groups ensured papers from our identified key journals were also highlighted. The Smart Group library enabled us to screen the most potentially relevant citations first. The other citations would remain unfiled but would still be available for a manual screen if time allowed.

The Smart Groups were tested before use—papers selected by the team using only manual screening were compared with the Smart Group papers (and those unfiled) over several weeks to ensure the search terms for each theme were retrieving the relevant papers.

Once established, the daily PubMed import could be deduplicated in the shared Endnote library and then copied into a Smart Groups library for screening. The ease of updating existing Smart Groups, and creating new ones, was of huge benefit. With the upsurge of Omicron‐variant papers, a new Smart Group ensured those papers with Omicron or B.1.1.529 in Title and/or Keyword were highlighted.

### Final decision‐making

**FIGURE 1:**
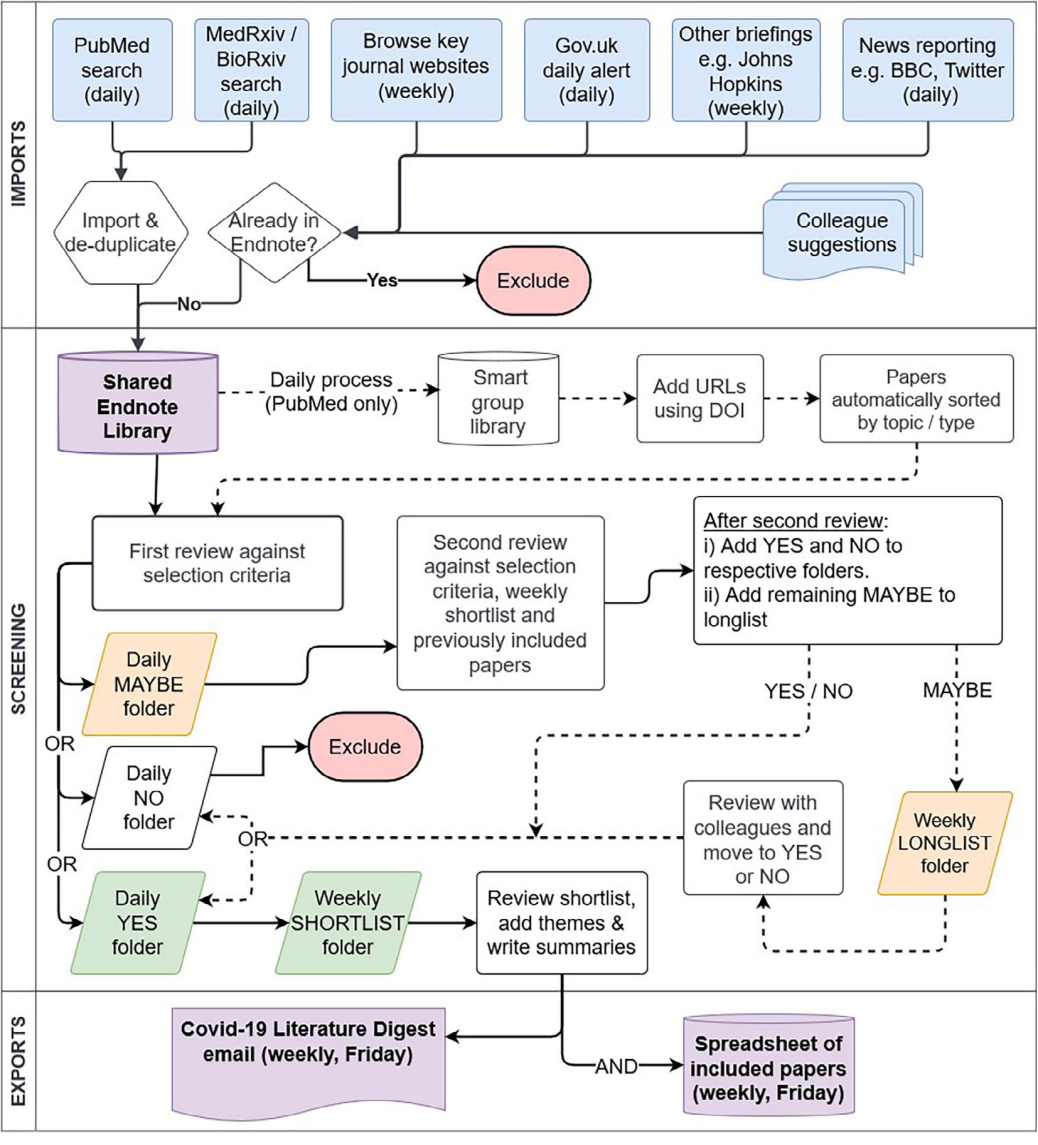
Production process for the Digest, as of March 2022. [Colour figure can be viewed at wileyonlinelibrary.com]

Each working day an Endnote folder was created with subfolders for daily PubMed and Preprint imports, as well as Yes, No and Maybe folders. Two additional subfolders were added each Friday: a Longlist to file outstanding ‘maybe’ decisions for reviewing with colleagues (which were discussed in a Thursday afternoon review meeting or via MS Teams chat), and a Shortlist to include all ‘yes’ decisions which were exported for creating the Digest on Friday afternoons. The Shortlist gave Digest team members an overview of what was included, allowing a final assessment, and helped avoid selecting and summarising very similar papers (see Figure 1).

The volume and publication speed of papers increased rapidly as publishers adapted, thereby increasing the difficulty of tracking duplicates and identifying published articles previously included in the Digest as preprints. For example, several versions of a preprint might be released with minor corrections, such as a change to the title which would prevent automatic deduplication by Endnote, and the period between preprint and published article sometimes narrowed from months to a few weeks. Occasionally, a preprint and its associated journal publication would be imported into the Endnote library during the same week. An Excel spreadsheet of previously included papers, containing bibliographic details and our bullet‐point summaries, was developed to identify duplicates in several ways, such as searching for keywords in the title or summary, or filtering by theme. This also enabled us to scan previously included citations on a particular topic, to establish how novel a new citation was. Bullet‐points could also be tailored to highlight previously unreported findings.

### Modifications and improvements to the Digest process over time

Changes to the methodology can be tracked through the 10 iterations of the SOP. In the first SOP (March 2020), our criteria included no exclusions by language or study type. With most research coming initially from China, this meant papers in Chinese languages with an English abstract could be included. As the volume of evidence grew, the inclusion criteria included English language only, and specified types of study to include or exclude.

Letters and comments were not automatically rejected; early in the pandemic many authors used letters to share their research data in order to shorten publication time (Teixeira da Silva et al., 2021). The importance of preprints during the COVID‐19 pandemic was recognised from the start and continued to be significant—the highest number of papers included in our Digests (249) during the first half of 2021 were posted on medRxiv. However, searching and screening preprints was a laborious process initially, as papers had to be screened online and downloaded one at a time. These platforms were gradually improved until batches of citations could be exported.

A disclaimer was added to all our included preprints, highlighting the absence of peer review, and later to highlight that the Digest was rapidly produced, selective and included non‐peer reviewed literature. If a published paper for inclusion had been previously included at the preprint stage, a note was added to the Digest text.

The papers in the Digest were categorised into six themes. By late April 2020, two extra categories enabled guidance, overviews and comments to be added without a bullet point summary. These initial themes remained stable until Spring 2021 when more categories (including one for Vaccines) were added.

### Study types and quality of included evidence

Due to the rapid nature of production and the volume of literature examined, detailed assessment of the quality of each paper included was not possible. However, we were able to narrow our selection criteria as the volume and quality of literature increased. Case studies or case series were excluded, as were animal studies, unless they presented novel research; overviews or non‐systematic reviews were omitted. Early systematic reviews built on few or non‐peer reviewed original studies were included at first as they were the only reviews available; we noted their limitations in the bullet points. Later, when a significant number of systematic reviews became available, we aimed to select only the higher quality ones. We developed a one‐page checklist that outlined the important information to look for when making a basic assessment of the quality of a systematic review. This included the dates of a search, search strategies, whether risk of bias or critical appraisal tools had been used or any limitations had been discussed, and if a PRISMA (Preferred Reporting Items for Systematic Reviews and Meta‐Analyses) flow diagram (http://prisma-statement.org/) was provided to show the flow of information through a review.

We also created a systematic reviews Smart Group in our Endnote library to aid identification. Systematic reviews were originally included under the relevant theme rather than as a separate category—to ensure these were highlighted, we added ‘Systematic Review’ as a new publication type. Where several systematic reviews with similar search dates on identical topics existed, we selected only the better quality one, and were able to check our Excel spreadsheet of previously included papers specifically for systematic reviews on a particular topic.

### User surveys

A total of three user surveys were conducted (in April and June 2020, and in January 2021). The aim of these surveys was to obtain feedback from subscribers to determine if the product remained valuable and inform necessary change. The third survey included questions asking how often people read the Digest, and whether they found the guest editorials of value.

A final evaluative survey (‘impact survey’; Appendix A) was conducted between January and February 2022 to explore how the Digest was used, the benefits to individuals and organisations, changes in usefulness as the pandemic progressed and to invite general feedback. It was conducted using Microsoft Forms and advertised in the header of three weekly Digest emails and an additional email to the mailing list.

## RESULTS

**FIGURE 2:**
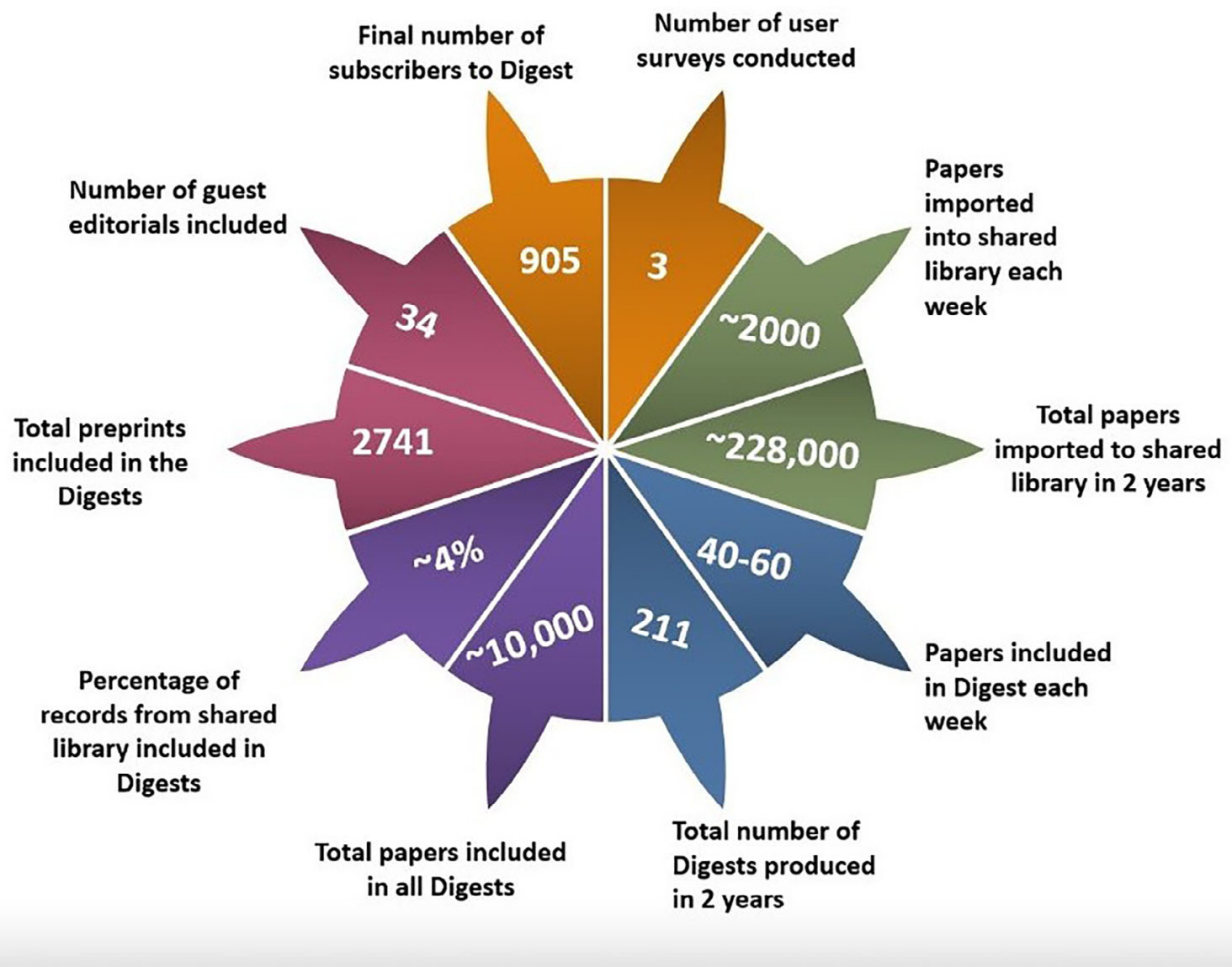
Infographic showing key numbers from Digest production. [Colour figure can be viewed at wileyonlinelibrary.com]

**FIGURE 3:**
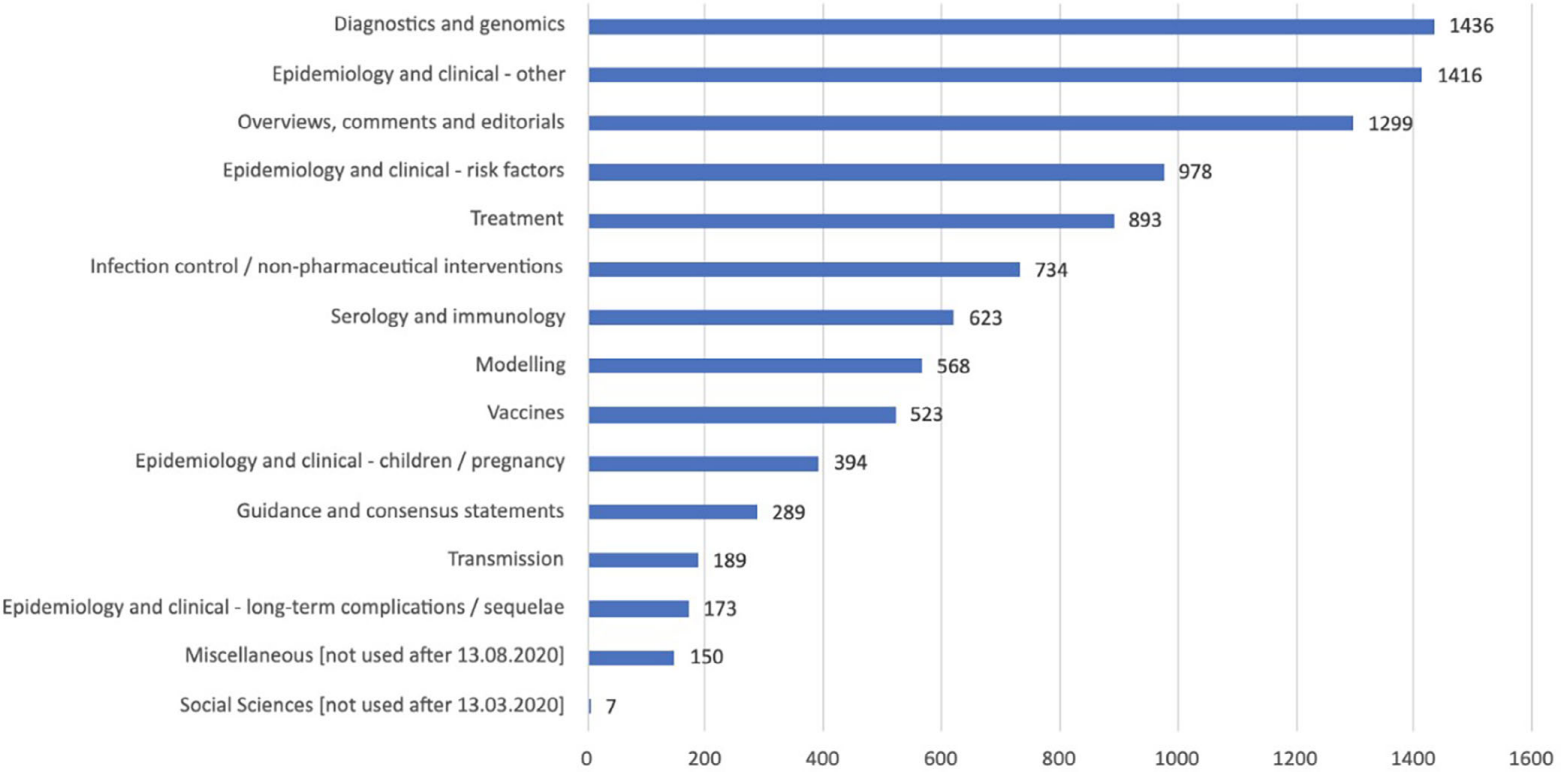
Chart showing the total number of papers included in the Digest by theme. [Colour figure can be viewed at wileyonlinelibrary.com]

Apart from the early pandemic period when evidence was sparse, around 2000 papers were imported into the shared Endnote library every week, totalling over 228,000 records imported in 2 years. Papers were screened using inclusion criteria, team discussion and later Endnote Smart Groups, with 40–60 papers selected, summarised and emailed to Digest subscribers each week (see an extract from the Digest in Appendix B, and a full example here: https://bit.ly/3sLUPIh). A total of around 10,000 research papers (4% of all records imported) were selected for inclusion in 211 Digests. Between June 2020 and May 2021, the Digest also included 34 guest editorials, where an invited editor highlighted and discussed 3 key papers. Figures 2 and 3 summarise some of the key numbers and totals from our Digest production.

### User survey results

The three user surveys obtained feedback which informed changes to the Digest. Our surveys in April and June 2020 supported a proposal to reduce the Digest from every weekday to three times a week. Our concern about how a proposal of reduced frequency would be accepted by subscribers, was negated by comments noting their lack of time to read a daily Digest. Following a survey suggestion to include guest editorials, between 22 June 2020 and 21 May 2021 we invited guest editors to highlight and review three papers of interest each week. The guest editor's review appeared at the top of the Friday digest, ‘If you only read three papers this week…’.

In the January 2021 survey [*n* = 121], 44% of respondents read the Digest once a week or less. The top suggestions for making it more sustainable were reducing the frequency to weekly; and decreasing the number of guest editorials. Both measures were implemented shortly afterwards, with a single Friday Digest and fortnightly guest editorials. Other comments stated how the Digest had reduced information overload, and the advantages of curating the wide variety of information into some sort of order.

Our 2021 survey also found that 20 (24%) respondents regularly forwarded the Digest to someone else, with a further 59 (71%) ‘sometimes’ forwarding it. This meant the number of people viewing the Digest was greater than the 905 subscribers. The largest number of subscribers were from UKHSA (30.2%), the National Health Service (NHS) (29.5%), and UK Government Departments/Agencies (13.5%).

### Impact survey results

The impact survey in January 2022 was completed by 148 subscribers. 102/148 respondents completed a voluntary question on job title—roles were varied, with high levels of consultants (n = 20) and library or information‐related roles (*n* = 12).The data suggested information in the Digest was used in several ways (Table 1), with the most popular option being ‘General interest/staying up‐to‐date’ (81.1%; *n* = 120).

**TABLE 1:** How have you personally used the information from the Digest?

Uses of Digest [multiple‐choice]	Total	Percentage of respondents (*n* = 148) selecting option
General interest/staying up‐to‐date	120	81.1%
Sharing information with colleagues	104	70.3%
For horizon scanning	47	31.8%
Informing own research/publications	40	27.0%
Continuing professional development (CPD)	34	23.0%
Developing/updating guidelines	34	23.0%
Informing patient care	24	16.2%
Informing a policy	23	15.5%
Producing a presentation	20	13.5%
Service development	12	8.1%
Other	3	2.0%
None of the above	2	1.4%

Key benefits of the Digest identified for individuals and organisations are detailed in Tables 2 and 3. Gaining new knowledge and saving time were the greatest individual benefits; the greatest organisational benefits were contributing to evidence‐based decision making and saving time.

**TABLE 2:** How has the Digest benefitted you personally?

Benefits to individuals (multiple‐choice)	Total	Percentage of respondents (*n* = 148) selecting option
Gained new knowledge	128	86.5%
Saved time	109	73.6%
Contributed to evidence‐based decision‐making	73	49.3%
Helped with educating others	58	39.2%
Increased my understanding of research studies	39	26.4%
Contributed to CPD	36	24.3%
Increased my interest in reading research	36	24.3%
Generated new ideas	32	21.6%
Changed my way of thinking	16	10.8%
Other	4	2.7%
None of the above	3	2.0%

**TABLE 3:** How do you think the Digest has benefitted your organisation?

Benefits to organisations (multiple‐choice)	Total	Percentage of respondents (*n* = 148) selecting option
Contributed to evidence‐based decision‐making	105	70.9%
Saved time	100	67.6%
Assisted with the production of evidence reviews and summaries	66	44.6%
Contributed to policy development	45	30.4%
Improved patient care	39	26.4%
Generated new ideas	37	25.0%
Contributed to service development	29	19.6%
Saved money	27	18.2%
Reduced risk	22	14.9%
None of the above	12	8.1%
Other	5	3.4%

**FIGURE 4:**
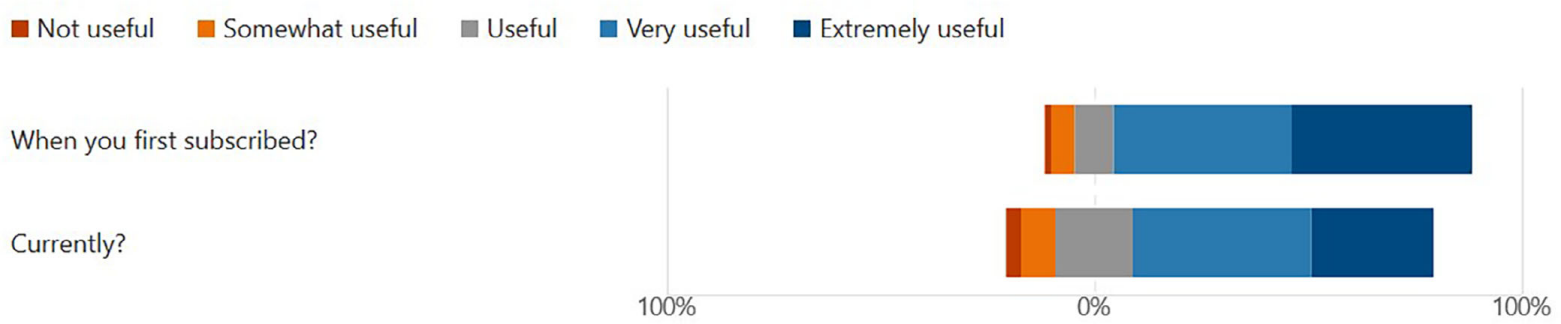
Perceived usefulness of Digest over time. [Colour figure can be viewed at wileyonlinelibrary.com]

Using a 1–5 Likert scale, respondents rated the usefulness of the Digest (i) when first subscribed and (ii) at the time of completing the impact survey. The data (Figure 4) suggests some reduction in usefulness over time, with the number rating the Digest as ‘Extremely useful’ reducing from 41.9% to 28.4%. Forty‐seven respondents completed an optional free text question giving reasons for their scores. Responses fell into two themes: (i) highlighting continuing usefulness or offering praise (*n* = 26), such as it saving people time and effort; and (ii) suggesting the Digest was less useful now (*n* = 21), as their concerns about COVID‐19 had gradually reduced over time or the evidence had become more ‘stable’.

The most popular alternatives to the Digest were ‘Reading journals/websites myself’ (75.7%; *n* = 112) and ‘Setting up an alert’ (37.2%; *n* = 55). Specific feedback on the Digest was received from 79 Digest readers (*n* = 62 via Impact Survey, *n* = 17 via email). Much of the feedback was about how people appreciated being reliably informed during the pandemic.

## DISCUSSION

At the beginning of the pandemic there was limited evidence available, but research output increased hugely and quickly (Teixeira da Silva et al., 2021). The rise of preprints on COVID‐19 was also rapid. Initially conceived and established by scientists, the Digest team consisted of volunteers seconded from UKHSA to help with the incident response. The Digest was later developed by members of the Knowledge and Library Services (KLS) and managed by a Healthcare Scientist, and finally the Digest team moved into the newly established COVID‐19 rapid evidence service (RES) (UKHSA COVID‐19 Rapid Evidence Service, 2022). By April 2021, a small dedicated team (consisting of two part‐time Knowledge and Evidence Specialists (KES) and one full time Assistant KES, supported and managed part‐time by one Senior Information Scientist) continued to organise the increasing volume of literature produced during the pandemic in a timely, responsive and structured way.

The Digest procedures constantly evolved as we sought quality improvement and time savings, becoming more streamlined and automated with the use of Endnote Smart Groups and addition of stricter inclusion criteria, although there was still a significant element of manual selection necessary. Human judgement was needed on whether to include a single case study, for example, because the findings may be novel and significant. Changes made in the shared Endnote library by one member of the team occasionally led to Sync conflicts, whereby the library would have two versions of the same citation record. Sync conflicts could be easily resolved, but they did become increasingly problematic as the total number of papers in the Endnote library approached 200,000. This problem was solved by ensuring working from the same folder simultaneously was reduced to a minimum, as well as reducing the size of the Endnote library by removing previously excluded papers older than 6 months.

The Digest process was also adapted to meet staff capacity restraints, for example by moving from daily to weekly publication as the amount of research grew. Capacity issues also meant that the Endnote Smart Group testing had to be completed by library colleagues outside the immediate Digest team, showcasing the difficulties of being able to step back and explore options to improve methodologies when in the midst of a response that required immediate deliverables. The SOP and inclusion criteria were regularly revised as COVID‐19 research progressed and study quality improved. The Digest was disseminated regularly in an easy‐to use output involving one click to access the full text (which was possible due to publishers allowing free access to COVID‐19 research articles from early 2020; Carr, 2020).

In order to facilitate knowledge transfer and reduce information overload, only a small proportion of the available evidence was included by the Digest team. Writing the bullet points required an understanding of evidence context as the team built up tacit knowledge. Joining the UKHSA's COVID‐19 RES in 2021 led to mutual benefits, as close creative connections were established between colleagues, who highlighted new articles, provided selection advice or commented on the quality of papers. The inclusion of guest editors from a wide spectrum of specialisms introduced perspectives from different teams, within our organisation and externally.

During production of the Digest we became aware of the extensive duplication of evidence reviews – for example, 30 systematic reviews on Remdesivir, a treatment for COVID‐19, were published in just one year, and many were already out of date (Elliott et al., 2021). In 2020, the Digest team saw more than 25 PROSPERO registrations (Centre for Reviews and Dissemination, 2022) for very similar reviews related to vitamin D as a treatment for COVID‐19. We also observed the growth of ‘living reviews’ which aim to reduce waste, duplication and out of date evidence synthesis (e.g., (Chou et al., 2022). These provide decision‐makers with more up to date evidence when research is rapidly emerging, but if living reviews are to become more widespread, more resources and a commitment to frequent updating will be required. The publication of rapid reviews (a type of knowledge synthesis in which the steps of a systematic review are streamlined, accelerated or omitted; Tricco et al., 2017) proliferated, and increasingly incorporated pre‐print papers as well as peer‐reviewed studies. It is possible that including preprints in reviews will continue beyond the pandemic period, certainly for newly emerging topics, so libraries and organisations will need to consider their current policy on preprints, and understand their pros and cons (Fry et al., 2019).

This process of developing a Digest outlined in this article has already been repeated successfully within UKHSA for production of a Monkeypox (MPX) Digest in June 2022—the COVID‐19 SOP was used with slight modifications for the new topic, and a fully formed and sustainable MPX Digest was launched within a week. We have also collated a list of 10 learning points to inform those who may wish to embark on a similar process in the future. We suggest that to set up a Digest of this nature, it is necessary to consult with all relevant experts from the beginning—involve library services, research or evidence teams, communications teams and relevant subject experts, and work together to develop the content strategy. The COVID‐19 Digest understandably had to be initiated at speed and without additional funding, and so this initial consultation and teamwork was sometimes lacking; scientific expertise within the team was also less available to us in the later stages of the pandemic.

### Lessons learned from producing the UKHSA COVID‐19 Literature Digest

1. *Learn (and share learning) from your successes and failures*—capture and record the learning (we arranged for an After Action Review with a facilitator in order to discuss what worked and didn't work which was circulated to managers); we also presented a poster to the European Association of Health Information and Libraries conference in June 2022 (*A rapid COVID‐19 evidence Digest was created by information specialists using a mix of automated and human processes*), wrote learning points and kept all previous iterations of the SOP
2. *Organisations are not always aware of their own research*—different departments do not necessarily know about related research within their own organisation; the Digest unexpectedly became a vehicle to highlight the latest research from UKHSA (and partners) to others within UKHSA
3. *The Digest had interest and impact beyond our own organisation*—initially requested internally, we promoted the Digest more widely following a request in our first user survey. Two thirds of our final subscribers were not from PHE/UKHSA; this wider impact was reflected in the feedback we received with organisations stating that our Digest was one of the most valuable resources available and helped them to focus on a small selection of more useful research
4. *Team communication is everything*—our Digest team worked closely, but remotely, under immense time pressure so good communication was key. This was greatly improved by use of MS Teams; daily online meetings and use of a chat function enabled collaboration, support and encouragement, and avoided isolation whilst working at home. Don't forget to have a laugh along the way
5. *Conduct user surveys to inform decision‐making and assess impact*—our surveys supported proposal to reduce the Digest frequency, leading to less stressful working patterns; discovering that many respondents gained new knowledge and saved their time by reading the Digest, was a great morale booster for the team
6. *The* ‘human touch’ *is important*—guest editorials ‘If you only read three papers this week…’ were added to the Digest; specialisms of guest editors included epidemiologist, microbiologist, behavioural scientist and modeller, giving a wide perspective; the editorials were well‐received with 48% in our January 2021 survey [*n* = 121] reading them regularly as a welcome ‘human addition’; however, these were very time‐consuming to organise and were eventually stopped in late 2021.
7. *Be flexible and responsive*—our Standard Operating Procedure underwent ten iterations in order to respond to changes in the evidence, feedback from our users and staff capacity; our ways of working were adapted
8. *Collaborative working with other teams led to unanticipated mutual benefits*—the Digest team joined the COVID‐19 RES, who were able to highlight specific papers of potential interest or assist with assessing quality; Digest team also identified new papers on topics for their rapid reviews
9. *Arranging cover in case of sickness or to take leave was a challenge*—taking annual leave, arranging other absences or finding time to train new staff was a major challenge for the Digest team; other staff did not have capacity, training or level of tacit knowledge to allow them to easily cover for absent Digest team members; the Digest was also understaffed for a significant period of time due to lack of funding; an early consideration of staffing requirements and skills is vital
10. *Take time to stand‐back and reflect*—build in time and flexibility to review processes and make adjustments; at first we did not have ‘the capacity to help our capacity’; when we were able to make just one change to become more efficient, it opened up space to make more changes

### Limitations

One limitation of our Digest process was that a routine process to highlight recalled papers was not in place (although we did monitor Retraction watch) (Retraction watch, 2020); therefore, we are only aware of one instance when a preprint included in the Digest was subsequently recalled. Secondly, papers were only included under one theme—COVID‐19 vaccines for children would be listed under ‘Epidemiology and clinical‐children/pregnancy’ but not ‘Vaccines’ for example—although the Digest was easily searchable. Thirdly, our spreadsheet of included papers may have been a useful resource for others if made more widely available. Fourthly, the need to limit number of papers included meant sometimes we had to choose between equally useful research on a particular topic when conclusions were similar. Fifthly, a Healthcare Scientist operated as Lead for the Digest production initially; their epidemiological and genetics knowledge proved useful for defining the themes, refining inclusion criteria and selecting studies, and this expertise was missed when they were seconded to another part of the response in early 2021. Finally, as highlighted in Lessons learned, our staff‐resourcing for the Digest was not sustainable long‐term, and we were fortunate to have no lengthy period of unplanned absence due to staff sickness or extended leave.

## CONCLUSIONS

Policy makers, researchers and those in public health required the latest evidence rapidly and frequently during the COVID‐19 pandemic. By necessity our Digest was a rapidly created product, which needed amending as the nature of the evidence and the available staff resources changed over time. The Digest was successfully produced within the limits of our available resource, although there was an immense workload and time pressure which was only sustainable for this vital pandemic period. Feedback from user surveys indicated many benefits of this knowledge transfer to individuals and organisations, including evidence‐based decision‐making. The learning from this Digest will inform evidence monitoring, selection and dissemination for future health crises.

## CONFLICT OF INTEREST STATEMENT

All above authors have no conflict of interest to declare.

## APPENDIX A

### A.1 IMPACT SURVEY

#### A.1.1. UKHSA COVID‐19 literature Digest—impact survey

Thank you for taking part in our survey. This is anonymous and should take 5–10 min to complete.

1. How have you personally used the information from the Digest? (Tick all that apply)
  ○ General interest/staying up‐to‐date
  ○ Sharing information with colleagues
  ○ Continuing professional development (CPD)
  ○ Informing own research/publications
  ○ Producing a presentation
  ○ Developing/updating guidelines
  ○ Service development
  ○ Informing a policy
  ○ For horizon scanning
  ○ Informing patient care
  ○ None of the above
  ○ Other [free text box provided]
2. How has the Digest benefitted you personally? (Tick all that apply)
  ○ Gained new knowledge
  ○ Changed my way of thinking
  ○ Saved time
  ○ Contributed to CPD
  ○ Helped with educating others
  ○ Contributed to evidence‐based decision‐making
  ○ Generated new ideas
  ○ Increased my interest in reading research
  ○ Increased my understanding of research studies
  ○ None of the above
  ○ Other [free text box provided]
3. How do you think the Digest has benefitted your organisation? (Tick all that apply)
  ○ Contributed to evidence‐based decision‐making
  ○ Saved time
  ○ Saved money
  ○ Reduced risk
  ○ Assisted with the production of evidence reviews and summaries
  ○ Improved patient care
  ○ Contributed to service development
  ○ Contributed to policy development
  ○ Generated new ideas
  ○ None of the above
  ○ Other [free text box provided]
4. How long have you subscribed to the Digest?
  ○ Under 3 months
  ○ 3–6 months
  ○ 6–12 months
  ○ over 12 months
5. How did you first learn/find out about the Digest?
  ○ Colleague forwarded me the Digest email
  ○ Saw it on UKHSA library webpage
  ○ Heard about it at a meeting
  ○ Heard about it from UKHSA knowledge and evidence specialists
  ○ Read about it in PHE Weekly
  ○ Read about it in another online communication (please specify in ‘Other’ box below)
6. On a scale of 1–5 (where 1 is not useful and 5 is extremely useful), how useful was this Digest
7. More information on your response to question 6 above (optional)
8. If this Digest did not exist, how would you find out about the types of COVID‐19 research studies contained in the Digest (tick all that apply)?
  ○ Colleague
  ○ Bulletins from my own organisation
  ○ Bulletins from other organisations (e.g., Johns Hopkins)
  ○ Reading journals/websites myself
  ○ Setting up an alert (e.g., Google News, journal content page)
  ○ Social media
  ○ Library
  ○ None of the above
  ○ Other [free text box provided]
9. How do you typically access the Digest?
  ○ Desktop/Laptop
  ○ Tablet (e.g., iPad)
  ○ Mobile phone
10. Have you ever had problems reading the Digest content or accessing the full text of included articles?
  ○ Yes—links to articles not working
  ○ Yes—could not access full text links as subscriber‐only content
  ○ Yes—format of Digest made it difficult to read as screen too small
  ○ No
  ○ Other [free text box provided]
11. In which organisation/sector do you work?
  ○ UK Health Security Agency (UKHSA)
  ○ Office for Health Improvement and Disparities (OHID)
  ○ NHS
  ○ UK Government Departments/Agencies (including Devolved Administrations)
  ○ UK Local Government
  ○ UK Higher Education
  ○ Non‐UK Government Departments/Agencies
  ○ Non‐UK Higher Education
  ○ Private sector (e.g., pharmaceuticals)
  ○ Other [free text box provided]
12. What role do you hold within your organisation/agency? (Optional)
13. Do you have any other comments or feedback about this Digest? (Optional)

## APPENDIX B

### B.1 SHORT EXTRACT FROM THE COVID‐19 LITERATURE DIGEST OUTPUT

Dear all,

Please find today's report below.

UKHSA's COVID‐19 Literature Digest has been produced since February 2020. A selection of our previous Digests can be found here. This resource aims to highlight a small selection of recent COVID‐19 papers that are relevant to UK settings, contain new data, insights or emerging trends. The Digest Team generate a report once per week (Fri). The reports include both preprints, which should be treated with caution as they are NOT peer‐reviewed and may be subject to change, and also research that has been subject to peer review and wider scrutiny. The Digest is very rapidly produced and does not claim to be a perfect product; the inclusion or omission of a publication should not be viewed as an endorsement or rejection by UKHSA. We do not accept responsibility for the availability, reliability or content of the items included in this resource.

To join our email distribution list, or to be removed, please send a request to covid.litdigest@phe.gov.uk. If you are interested in papers relating to behaviour and social science please contact covid19.behaviouralscience@phe.gov.uk to sign up to receive the Behavioural Sciences Weekly Report.

Best wishes,

Emma Farrow, James Robinson

*On behalf of the UKHSA COVID‐19 Literature Digest Team*

*____________________________________________*

*Report for 04.02.2022* (please note that papers that have *NOT been peer‐reviewed* are highlighted in red).

Sections:

Serology and immunology
Vaccines
Diagnostics and genomics
Epidemiology and clinical—children and pregnancy
Epidemiology and clinical—long‐term complications/sequelae
Epidemiology and clinical—risk factors
Epidemiology and clinical—other
Infection control/non‐pharmaceutical interventions
Transmission
Treatment
Modelling
Overviews, comments and editorials (no digest)

Serology and immunology

**Table:**

Publication Date	Title/URL	Journal/Article type	Digest
2 February 2022	T‐cell reactivity to the SARS‐CoV‐2 Omicron variant is preserved in most but not all individuals	Cell/Article	• Authors identify a subset of individuals (∼21%) with a >50% reduction in T‐cell reactivity to the Omicron spike. • Findings support vaccine approaches that induce robust T‐cell responses targeting both variant spike and non‐spike antigens to overcome SARS‐CoV‐2 evolution.

Vaccines

**Table:**

Publication Date	Title/URL	Journal/Article type	Digest
1 February 2022	Protection by 4th dose of BNT162b2 against Omicron in Israel	medRxiv (non‐peer reviewed)/Article	• Authors draw on Israeli Ministry of Health data; 1,138,681 persons ≥60 years old eligible for fourth dose. • Rates of confirmed Covid‐19 and severe illness for persons ≥12 days after fourth dose were lower by factor of (i) 2.0 and 4.3 than three dose group; (ii) 1.9 and 4.0 than 3–7 days after 4th dose group. • Those who had not yet received a fourth dose had almost twice the number of risk days compared to persons ≥12 days after fourth dose (7.6 millions compared to 3.4 millions), more confirmed infections (42,693 vs. 9071) and severe cases (195 vs. 13).
27 January 2022	Persistence of protection against SARS‐CoV‐2 clinical outcomes up to 9 months since vaccine completion: a retrospective observational analysis in Lombardy, Italy	Lancet Infect Dis/Article	• Italian retrospective observational study • 5,351,085 individuals (≥12 years) who received complete vaccination from 17.01.2021 to 31.07.2021, were followed up from 14 days after vaccine completion until 20 October 2021 • Outcomes measured include infection rates and severe illness amongst vaccinated (vs. unvaccinated) • Findings include increase in infection rates and serious illness remain low but gradual increase in months following vaccination • Associated comment: https://doi.org/10.1016/s1473‐3099(22)00003‐2

## References

[hir12523-bib-0001] BBC . (2022). BBC health . https://www.bbc.co.uk/news/health

[hir12523-bib-0002] Brierley, L. (2021). Lessons from the influx of preprints during the early COVID‐19 pandemic. The Lancet Planetary Health, 5(3), e115–e117. 10.1016/S2542-5196(21)00011-5 33713612

[hir12523-bib-0003] Callaway, E. (2020). Will the pandemic permanently alter scientific publishing? Nature, 582(7811), 167–168. https://www.nature.com/articles/d41586-020-01520-4 10.1038/d41586-020-01520-4 32504015

[hir12523-bib-0004] Carr, D. (2020). Publishers make coronavirus (COVID‐19) content freely available and reusable . https://wellcome.org/press-release/publishers-make-coronavirus-covid-19-content-freely-available-and-reusable

[hir12523-bib-0005] Centre for Reviews and Dissemination . (2022). PROSPERO. International prospective register of systematic reviews. https://www.crd.york.ac.uk/prospero/

[hir12523-bib-0006] Chou, R. , Dana, T. , & Jungbauer, R. (2022). Update Alert 8: Masks for Prevention of Respiratory Virus Infections, Including SARS‐CoV‐2, in Health Care and Community Settings. Annals of Internal Medicine, 175(9), W108–W109. 10.7326/L22-0272 35878407 PMC9380719

[hir12523-bib-0007] Clarivate . (2022). Endnote . https://endnote.com/

[hir12523-bib-0008] Cold Spring Harbor Laboratory, Yale University, & BMJ . (2020a). COVID‐19 SARS‐CoV‐2 preprints from medRxiv and bioRxiv. https://connect.medrxiv.org/relate/content/181

[hir12523-bib-0009] Cold Spring Harbor Laboratory, Yale University, & BMJ . (2020b). medRxiv*. The preprint server for health sciences* . https://www.medrxiv.org/

[hir12523-bib-0010] Elliott, J. , Lawrence, R. , Minx, J. C. , Oladapo, O. T. , Ravaud, P. , Tendal Jeppesen, B. , Thomas, J. , Turner, T. , Vandvik, P. O. , & Grimshaw, J. M. (2021). Decision makers need constantly updated evidence synthesis. Nature, 600(7889), 383–385. 10.1038/d41586-021-03690-1 34912079

[hir12523-bib-0011] Epistemonikos . (2020). COVID‐19 L·OVE . https://app.iloveevidence.com/loves/5e6fdb9669c00e4ac072701d

[hir12523-bib-0012] Fry, N. K. , Marshall, H. , & Mellins‐Cohen, T. (2019). In praise of preprints. Microb Genom, 5(4), e000259. 10.1099/mgen.0.000259 PMC6521583 30938670

[hir12523-bib-0013] Glasziou, P. P. , Sanders, S. , & Hoffmann, T. (2020). Waste in covid‐19 research. BMJ, 369, m1847. 10.1136/bmj.m1847 32398241

[hir12523-bib-0015] Henrina, J. , Lim, M. A. , & Pranata, R. (2021). COVID‐19 and misinformation: how an infodemic fuelled the prominence of vitamin D. British Journal of Nutrition, 125(3), 359–360. 10.1017/S0007114520002950 32713358 PMC7443564

[hir12523-bib-0016] Jung, R. G. , Di Santo, P. , Clifford, C. , Prosperi‐Porta, G. , Skanes, S. , Hung, A. , Parlow, S. , Visintini, S. , Ramirez, F. D. , Simard, T. , & Hibbert, B. (2021). Methodological quality of COVID‐19 clinical research. Nature Communications, 12(1), 943. 10.1038/s41467-021-21220-5 PMC7878793 33574258

[hir12523-bib-0017] National Library of Medicine . (2020). LitCovid. https://www.ncbi.nlm.nih.gov/research/coronavirus/ 10.1080/15360280801989377 28792816

[hir12523-bib-0018] Pearson, H. (2021). How COVID broke the evidence pipeline. Nature, 593(12) https://www.nature.com/articles/d41586-021-01246-x, 182–185. 33981057 10.1038/d41586-021-01246-x

[hir12523-bib-0019] Retraction Watch . (2020). Retraction watch. Retracted coronavirus (COVID‐19) papers . https://retractionwatch.com/retracted-coronavirus-covid-19-papers/

[hir12523-bib-8001] Sage (Scientific Advisory Group for Emergencies) Committee . (2022). Scientific evidence supporting the government response to coronavirus (COVID‐19). https://www.gov.uk/government/organisations/scientific-advisory-group-for-emergencies

[hir12523-bib-0020] Teixeira da Silva, J. A. , Tsigaris, P. , & Erfanmanesh, M. (2021). Publishing volumes in major databases related to Covid‐19. Scientometrics, 126(1), 831–842. 10.1007/s11192-020-03675-3 32904414 PMC7454548

[hir12523-bib-0021] Tricco, A. C. , Langlois, E. V. , Straus, S. E. , & Alliance for Health Policy Systems and Research, & World Health Organization . (2017). *Rapid reviews to strengthen health policy and systems: a practical guide* (9789241512763). CC BY‐NC‐SA 3.0 IGO). https://apps.who.int/iris/handle/10665/258698

[hir12523-bib-0022] UK Health Security Agency . (2021). UK Health Security Agency launches with a relentless focus on keeping the nation safe . Retrieved September 30, 2022 from https://www.gov.uk/government/news/uk-health-security-agency-launches-with-a-relentless-focus-on-keeping-the-nation-safe

[hir12523-bib-0023] UKHSA COVID‐19 Rapid Evidence Service . (2022). UKHSA COVID‐19 rapid reviews . https://ukhsalibrary.koha-ptfs.co.uk/covid19rapidreviews/

